# *Beta-3-adrenergic Receptor* rs4994 Polymorphism Is a Potential Biomarker for the Development of Nonalcoholic Fatty Liver Disease in Overweight/Obese Individuals

**DOI:** 10.1155/2019/4065327

**Published:** 2019-12-17

**Authors:** Yuki Sakamoto, Kentaro Oniki, Naoki Kumagae, Kazunori Morita, Koji Otake, Yasuhiro Ogata, Junji Saruwatari

**Affiliations:** ^1^Division of Pharmacology and Therapeutics, Graduate School of Pharmaceutical Sciences, Kumamoto University, Kumamoto 862-0973, Japan; ^2^Japanese Red Cross Kumamoto Health Care Center, Kumamoto 861-8528, Japan

## Abstract

Nonalcoholic fatty liver disease (NAFLD) is one of the most prevalent chronic liver diseases. Obesity is the most common and well-established risk factor for NAFLD, but there are large interindividual differences in the relationship between weight status and the development of NAFLD. Beta-3-adrenergic receptor (ADRB3) plays a key role in the development of visceral obesity and insulin resistance; however, the effect of *ADRB3* polymorphisms on the risk of NAFLD remains unclear. We investigated whether or not a common rs4994 polymorphism (T190C) in the *ADRB3* gene is associated with the risk of NAFLD through an increase in the body mass index (BMI) among the general population. We performed cross-sectional and longitudinal analyses in a total of 591 Japanese health screening program participants. Among the overweight or obese subjects, but not normal-weight subjects, individuals with the C/C genotype had a higher risk of developing NAFLD in comparison to those with other genotypes in the cross-sectional analysis (odds ratio: 4.40, 95% confidence interval (CI): 1.08–17.93). Meanwhile, the receiver operating characteristic curve indicated that the association between an increase in the BMI and the presence of NAFLD in subjects with the C/C genotype (area under the curve: 0.91, 95% CI: 0.78–1.00) was more pronounced in comparison to subjects with other genotypes. These above-described findings were verified by the analyses using a replicated data set consisting of 5,000 random samples from original data sets. Furthermore, among the 291 subjects for whom longitudinal medical information could be collected and who did not have NAFLD at baseline, the Cox proportional hazard model also confirmed that overweight or obese status and the C/C genotype were concertedly related to the increased risk of NAFLD development. These results suggest that genotyping the *ADRB3* rs4994 polymorphism may provide useful information supporting the development of personalized BMI-based preventive measures against NAFLD.

## 1. Introduction

Nonalcoholic fatty liver disease (NAFLD), considered to be a hepatic phenotype of metabolic syndrome, is one of the most prevalent chronic liver diseases, and the prevalence has been increasing worldwide [1, 2]. NAFLD is closely associated with not only the risk of advanced liver disease but also metabolic complications, including type 2 diabetes, cardiovascular disease, and chronic kidney disease [3]. Obesity is the most common and well-established risk factor for NAFLD [2]. However, NAFLD has also been observed in nonobese subjects, especially in Asian populations [4–7]. The population background (e.g., genetic polymorphisms, lifestyle, age, gender, and underlying diseases) plays substantial roles in the association between weight gain and the development of NAFLD [4–7], and assessing these factors can be useful for the early identification of individuals with a high risk of developing NAFLD and the further development of targeted prevention and treatment programs for high-risk populations [8].

Beta-3-adrenergic receptor (ADRB3) is mainly expressed in the visceral adipose tissues and plays an important role in the enhancement of thermogenesis in brown adipocytes and the promotion of lipolysis in white adipose tissues, all of which are induced by the activation of the sympathetic nerves [9–11]. ADRB3 mediates the catecholamine-induced activation of adenylate cyclase through the action of G proteins and contributes to variations in energy expenditure and body fat distribution [12, 13]. A previous study of ADRB3 knockout mice indicated that ADRB3 plays a fundamental role in the regulation of body weight by mediating the adrenergic stimulation of white adipose tissue lipolysis, particularly when the adipocytes are loaded with triglycerides [14]. In adult mice with the adipocyte-specific deletion of Berardinelli-Seip congenital lipodystrophy type 2 (i.e., Bscl2), the expression of ADRB3 protein was downregulated in mature adipose tissue, which increased the development of high-fat diet-induced hepatic steatosis despite reducing adrenergic-stimulated lipolysis [15].

To date, several polymorphisms have been identified in the *ADRB3* gene. Among them, a number of studies have focused on a common *ADRB3* rs4994 polymorphism (T190C, Trp64Arg) to investigate the genetic effect on the development of obesity [16–19]. In the case of T190C polymorphism, C allele carriers were found to have a high risk of abdominal obesity [16, 17]; the polymorphism has also been linked to the increased development of insulin resistance [16]. Although the association of the polymorphism with overweight and insulin resistance is controversial and likely dependent on ethnicity, recent meta-analyses showed that the *ADRB3* rs4994 polymorphism is significantly associated with the body mass index (BMI) and type 2 diabetes, especially in East Asians [18, 20, 21]. In Japanese subjects with a moderate degree of obesity (BMI 22–26.4 kg/m^2^), an rs4994 C allele carrier status was associated with visceral obesity and lower serum triglyceride levels [22]. rs4994 C allele carriers were shown to lower lipolytic activity in comparison to subjects with T/T genotype, and this polymorphism may therefore be associated with the accumulation of triglycerides in adipocytes, thereby leading to the accumulation of visceral fat and the development of insulin resistance [22, 23].

The copresence of metabolic abnormalities associated with the rs4994 C allele is widely considered to be the pathogenesis of NAFLD [24, 25]. Insulin resistance in adipose tissue can dysregulate adipose tissue lipolysis and thus increase the flux of fatty acids from adipocytes to the liver [26], and insulin resistance-induced hyperglycemia/hyperinsulinemia promotes hepatic *de novo* lipogenesis [27], both of which lead to the development of NAFLD [24, 25]. Therefore, it is hypothesized that the rs4994 polymorphism may be associated with the development of NAFLD directly and/or indirectly. Actually, several cross-sectional studies of Japanese populations have implicated the *ADRB3* rs4994 polymorphism in liver dysfunction, including nonalcoholic steatohepatitis (NASH) [28, 29]. Among normal-weight subjects (BMI 19.8–24.2 kg/m^2^), an *ADRB3* rs4994 C allele carrier status was associated with elevated alanine aminotransferase (ALT) levels [28]. Moreover, the C allele frequency in NASH patients was significantly higher than that in control subjects, and among the NASH patients, a C allele carrier status was associated with an increased BMI and higher levels of hypertriglyceridemia and hyperinsulinemia [29]. Nevertheless, how this polymorphism affects the association between the weight status and the development of NAFLD as well as whether or not it is a potential biomarker for screening individuals for high risk of NAFLD remains unclear.

Based on this information, the current study investigated whether or not the rs4994 polymorphism in the *ADRB3* gene is associated with the development of NAFLD through an increase in the BMI among the general population. Because the rs4994 polymorphism is common and potentially functional [18, 20, 21], especially in Asian populations (e.g., the allele frequency in Japanese is approximately 20% [17]), this study investigated the genetic effects in Japanese subjects who participated in a health screening program.

## 2. Materials and Methods

### 2.1. Subjects and Study Protocol

A cross-sectional analysis was conducted in 591 Japanese subjects (male, *n* = 350; female, *n* = 241; mean age, 64.5 ± 12.1 years) who were consecutively recruited from a group of participants in a health screening program at the Japanese Red Cross Kumamoto Health Care Center between May 2003 and April 2012. Among these subjects, a retrospective longitudinal analysis with 5.3 ± 1.2 (mean ± standard deviation) years of follow-up was performed in the 291 subjects (male, *n* = 172; female, *n* = 119; mean age at baseline, 67.8 ± 5.8 years) who did not have NAFLD at baseline and for whom longitudinal medical information was able to be collected from January 1, 2006, to April 30, 2012. Ongoing or recent alcohol consumption was calculated using the following equations: pure  alcohol mass (g) = volume (mL) × alcohol by volume × volumetric mass density (0.8 g/mL). All subjects were nondrinkers or moderate drinkers (<30 g/day of alcohol for males and <20 g/day for females) and were hepatitis B and C virus-negative, according to the previously reported practical guidelines for NAFLD [2].

This study protocol was approved by the institutional ethics committees of the Faculty of Life Sciences, Kumamoto University, and the Japanese Red Cross Kumamoto Health Care Center (Approval No. 169). All subjects provided their written informed consent only once prior to enrollment in the study. The study was performed in accordance with Ethical Guidelines for Epidemiological Research in Japan.

### 2.2. Measurements

Normal-weight, overweight, and obese statuses [30] were defined as BMI < 23 kg/m^2^, ≥23 kg/m^2^ to <27.5 kg/m^2^, and ≥27.5 kg/m^2^, respectively, according to the recommendations of the American Diabetes Association regarding the screening of Asians who are at risk for type 2 diabetes [31]. Hepatic ultrasonography was used to diagnose fatty liver disease (FLD). The FLD was diagnosed based on the following four criteria: (1) a diffused hyperechoic echotexture (bright liver), (2) an increased echo texture in comparison to that of the kidneys, (3) vascular blurring, and (4) deep attenuation [20]. The diagnosis of FLD was made by the radiographer. Medical doctors (authors: K.O. and Y.O.) then reviewed the images to evaluate the accuracy and reproducibility of the diagnosis. The blood pressure (BP) was measured 3 times using a standard mercury sphygmomanometer after a 5 min rest with the subject in the sitting position, and the average of the measurements was used. Hypertension was defined as a systolic blood pressure (BP) ≥ 140 mmHg, a diastolic BP ≥ 90 mmHg, or a history of hypertension [32]. Dyslipidemia was defined as a value of triglycerides (TG) ≥ 150 mg/dL, high-density lipoprotein cholesterol (HDL_C) < 40 mg/dL, low-density lipoprotein cholesterol (LDL_C) ≥ 140 mg/dL, or a history of dyslipidemia [33]. Diabetes defined as a fasting plasma glucose (FPG) level of ≥126 mg/dL or a casual or 2 h glucose level of ≥200 mg/dL after a 75 g OGTT and a hemoglobin A1c (HbA1c) level of ≥6.5% or a history of diabetes [34]. FPG (mg/dL) and other biochemical examinations of blood were determined from blood samples after an approximately 12 h fast using the standard methods of the Japan Society of Clinical Chemistry. Fasting serum insulin (FSI) (*μ*U/mL) was measured using an insulin enzyme-linked immunosorbent assay (ELISA) kit (Human Insulin ELISA Kit; Mercodia, Uppsala, Sweden). The homeostasis model assessment for insulin resistance (HOMA-IR) values were calculated using the following equation: HOMA − IR = (FSI × FPG)/405 [35]. Information regarding the subjects' alcohol intake and smoking habits was obtained via face-to-face interviews with health care providers using the following questions: “How often do you drink? (1. Never, 2. 1-2 times per week, 3. 3-4 times per week, 4. 5-6 times per week, 5. Every day)” “How many units of alcohol do you drink on a typical day when you are drinking?” “Are you a smoker?” “How many cigarettes a day do you smoke?” “When did you start smoking?”

### 2.3. Genotyping

Genomic DNA was extracted from the whole blood using a DNA purification kit (FlexiGene DNA kit; QIAGEN, Hilden, Germany). The *ADRB3* rs4994 polymorphism was detected using a real-time TaqMan allelic discrimination assay (assay no. C_2215549_20; Applied Biosystems, Waltham, MA, USA) [36, 37] in accordance with the manufacturer's protocol. To ensure the genotyping quality, we included DNA samples as internal controls, hidden samples of a known genotype, and negative controls (water).

### 2.4. Statistical Analyses

The data are expressed as the median (range) or proportion for categorical variables. Since no continuous variables were normally distributed, the Kruskal Wallis test was used for comparisons. Fisher's exact test was used for comparisons of categorical variables. The odds ratio (OR) and 95% confidence interval (CI) for the risk of NAFLD were calculated using bivariable or stepwise, forward selection multivariable logistic regression models. To consider the subject weight status carefully in our analyses, we also investigated the interaction of the *ADRB3* genotype and weight status in a multivariable logistic regression model. A multiple regression analysis was performed to compare the differences in the BMI and HOMA-IR values among the *ADRB3* genotypes. A receiver operating characteristic (ROC) curve analysis was performed to assess the relationship between an increase in BMI and the presence of NAFLD with calculations of the area under the curve (AUC). We tried to validate the findings observed in the original data set using the same statistical procedures as described above with a replicated data set generated from 5,000 randomly sampled subjects from the original data set with replacement and stratification according to the study population to ensure a representative study population distribution using the individual as the sampling unit. Structural equation modeling [38] was used to assess the indirect effects of the *ADRB3* genotype on the risk of developing NAFLD. This modeling consisted of two parts: (1) the selection of the predictors and mediators influencing the risk of NAFLD and (2) a path analysis of the relationships among the predictors, mediators, and risk of NAFLD. In the present study, we incorporated the cofactors of the prediction models for the risk of NAFLD into a structural equation model as predictors or mediators. The hypothesized models were tested against alternative models to assess the goodness of fit. The goodness of fit on the structural equation modeling was evaluated based on the following criteria: goodness of fit index (GFI) > 0.90, adjusted goodness of fit index (AGFI) > 0.90, and root mean square error of approximation (RMSEA) < 0.05. To verify the relationship between the *ADRB3* genotype and the development of NAFLD, the NAFLD-free survival according to the combination of the *ADRB3* genotype and the weight status at baseline was estimated using the Kaplan-Meier survival curves from January 1, 2006, to April 30, 2012, among the subjects who did not have NAFLD at baseline. A comparison of the cumulative incidence between the groups was carried out using the log rank test. A multivariable-adjusted hazard ratio (HR) was also calculated using a Cox proportional hazard model adjusted for age, HOMA-IR, and dyslipidemia. A value of *p* < 0.05 was considered to be statistically significant. Multiple comparisons were corrected using Bonferroni's method, and values of *p* < 0.05/*n* were considered to be statistically significant after correcting the number of comparisons made. One author (Y.S.) performed all statistical analyses, and two authors (K.O. and J.S.) reviewed the raw data and the results of the statistical analyses. The statistical analyses and structural equation modeling were performed using the SPSS software program (version 23.0; IBM Japan Inc. Tokyo, Japan) and the SPSS Amos software program (version 23.0; IBM Japan Inc.), respectively.

## 3. Results

The allele frequency of the *ADRB3* rs4994 C allele was 21.0% in a total of 591 study subjects. The frequencies of the *ADRB3* rs4994 T/T, T/C, and C/C genotypes were 61.9%, 34.2%, and 3.9%, respectively, and were consistent with the Hardy-Weinberg equilibrium (*p* > 0.05). Although the prevalence of obesity tended to be higher in the C/C genotype than in the T/C and T/T genotypes, the association did not reach statistical significance (Table 1). The BMI values and the prevalence of overweight did not differ among the genotypes (Table 1). Among all subjects, 119 (20.1%) subjects were diagnosed with NAFLD; the prevalence did not differ between the C/C genotype and the T allele carriers (30.4% vs. 19.7%, *p* = 0.29).

We performed bivariable and multivariable logistic regression analyses to determine the association between subjects' characteristic, including the *ADRB3* genotype, and the risk of NAFLD (Table 2). All significant covariates found in bivariable analyses were included in a multivariable stepwise forward selection logistic regression model with the *ADRB3* genotype. In a multivariable logistic regression analysis of all subjects, although the risk of NAFLD in subjects with the C/C genotype tended to be higher than that in subjects with the T/T genotype, the association did not reach statistical significance (Table 2). We then determined the effect of the *ADRB3* genotype on the development of NAFLD, while carefully considering the weight status. Since the number of obese individuals was too small (*n* = 34), we combined them with overweight individuals (overweight/obese subjects). The *ADRB3* genotype and weight status (normal-weight or overweight/obese) tended to have interactive effects on the risk of NAFLD (*p* = 0.08); thus, multivariable analyses were performed separately in normal-weight and overweight/obese subjects. A significant association between the C/C genotype and a high prevalence of NAFLD was observed in overweight/obese subjects, but not in normal-weight subjects (Table 3). When obese individuals were excluded from the overweight/obese subjects, the association between the C/C genotype and the risk of NAFLD was also observed (Table 3). Among the obese subjects only, the number of subjects was too small (*n* = 34) to calculate this association. The clinical characteristics did not differ markedly among the genotypes in either normal-weight or overweight/obese subjects (Tables 4 and 5). In multiple linear regression analyses of overweight/obese subjects, individuals with the C/C genotype tended to be associated with a high BMI and HOMA-IR values (Table 6). These associations were not observed in normal-weight subjects (Table 6).

**Figure 1:**
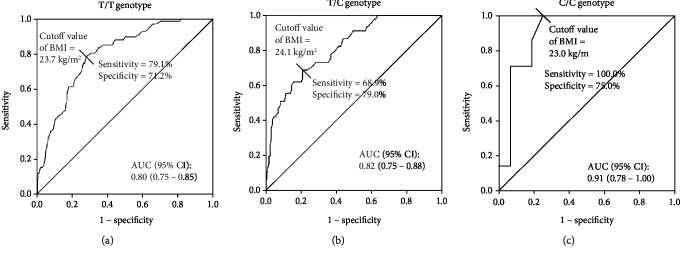
The ROC curves for the presence of NAFLD based on the BMI in each *ADRB3* genotype. The curves of the subjects with the *ADRB3* rs4994 T/T (*n* = 366) (a), T/C (*n* = 202) (b), and C/C (*n* = 23) (c) genotypes are shown separately. ROC: receiver operating characteristic; NAFLD: nonalcoholic fatty liver disease; BMI: body mass index; ADRB3: beta-3-adrenergic receptor; AUC: area under the curve; CI: confidential interval.

Second, we created an ROC curve to determine the relationship between the BMI and the presence of NAFLD among all study subjects for each *ADRB3* genotype. An increased BMI was significantly associated with the presence of NAFLD, especially in subjects with the C/C genotype (cut-off value of BMI = 23.0 kg/m^2^, Figure 1). The subjects with the C/C genotype tended to show larger AUC values than those with the T/T or T/C genotype (Figure 1), although the number of subjects with the C/C genotype was too small (*n* = 23) compared to the other two groups.

**Figure 2:**
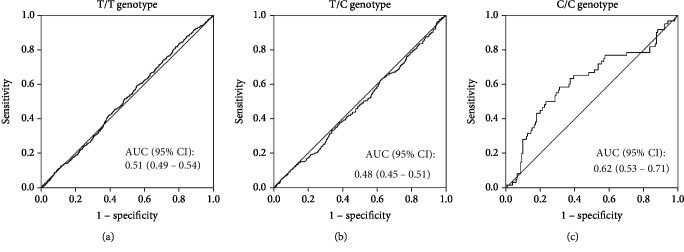
The ROC curves for the presence of NAFLD based on the BMI in each *ADRB3* genotype, using 5,000 randomly sampled data sets. The curves of the subjects with the *ADRB3* rs4994 T/T (*n* = 3084) (a), T/C (*n* = 1706) (b), and C/C (*n* = 210) (c) genotypes are shown separately. ROC: receiver operating characteristic; NAFLD: nonalcoholic fatty liver disease; BMI: body mass index; ADRB3: beta-3-adrenergic receptor; AUC: area under the curve; CI: confidential interval.

Third, we performed the same analyses using 5,000 replicated randomly sampled data sets. The clinical characteristics of the data sets are shown in Table 7. As a result, we confirmed that the risk of NAFLD in subjects with the *ADRB3* C/C genotype was significantly higher than that in T allele carriers among overweight/obese subjects but not normal-weight subjects (Table 8, Figure 2), although the number of subjects with the C/C genotype was still relatively small (*n* = 210) compared to the other two groups.

**Figure 3:**
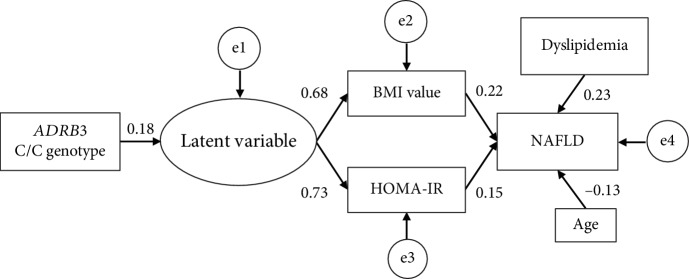
The structural equation modeling diagram of NAFLD and the *ADRB3* genotype in overweight/obese subjects (*n* = 285). Lines with numbers indicate significant paths with standardized partial regression (*β*) coefficients (*p* < 0.05). e1, e2, e3, and e4 indicate random effects. Arrows indicate an association between two factors. The *β* values ranged from -1 to 1, with a positive value representing a positive correlation and a negative value representing a negative correlation.

Fourth, to evaluate the relationships among the *ADRB3* genotype, the prevalence of NAFLD, and their related covariates in more detail, we performed a structural equation modelling approach using the original data set. As we were unable to identify a significant model in all subjects, we constructed models separately in normal-weight and overweight/obese subjects. Because the parameters (e.g., standardized partial regression coefficients) did not converge in normal-weight subjects (*n* = 306), the structural equation model incorporating the *ADRB3* genotype could not be developed. In overweight/obese subjects (*n* = 285), the effect of the *ADRB3* C/C genotype is modeled directly on the latent variable affecting an increased BMI and HOMA-IR and indirectly on the risk of developing NAFLD (Figure 3). The *p* value for the model fit to a *χ*^2^ (12.4, degrees of freedom = 8) was 0.135, and the GFI, AGFI, and RMSEA were 0.985, 0.961, and 0.045, respectively. Taken together, these fitness statistics indicated a good fit for the structural equation model in overweight/obese subjects. The *ADRB3* C/C genotype was associated with a high BMI and HOMA-IR through a latent variable (Figure 3), which may be the decreased lipolytic activity or thermogenesis. However, the *ADRB3* C/C genotype was not directly associated with the risk of developing NAFLD (Figure 3). A high BMI and HOMA-IR were directly associated with the risk of NAFLD (Figure 3).

**Figure 4:**
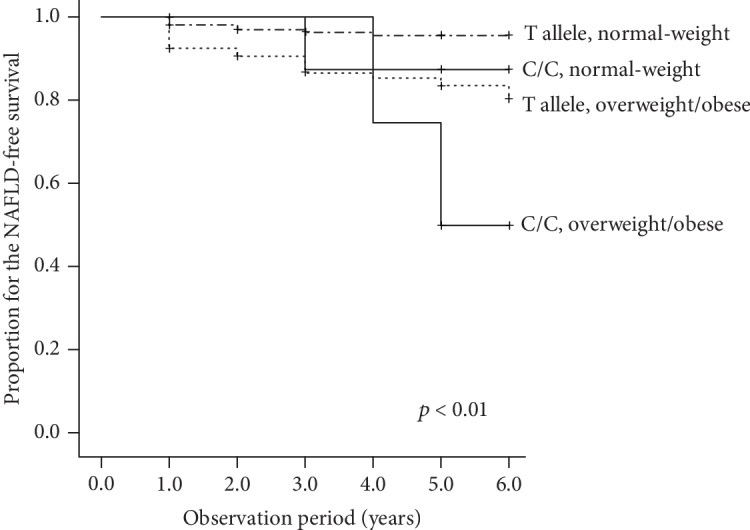
Kaplan-Meier curves for the NAFLD-free survival in the 291 subjects for whom longitudinal medical information could be collected and who did not have NAFLD at baseline. The comparison of the cumulative incidence among groups was carried out using the log rank test.

Finally, we performed a longitudinal analysis to verify the relationship between the *ADRB3* genotype and the development of NAFLD in overweight/obese subjects. The prevalence of NAFLD at the endpoint was 10.0% among the 291 subjects who did not have NAFLD at baseline. The overweight/obese subjects with the *ADRB3* C/C genotype exhibited a higher incidence of NAFLD than normal-weight subjects with the T/T or T/C genotypes (*p* < 0.01) (Figure 4). In a multivariable Cox proportional hazard model, overweight/obese subjects with the C/C genotype were significantly associated with the incidence of NAFLD (Table 9).

## 4. Discussion

This is, to the best of our knowledge, the first study to find a possible association between the *ADRB3* rs4994 polymorphism and the development of NAFLD through an increase in BMI and insulin resistance. ADRB3 plays significant roles in the enhancement of thermogenesis in brown adipocytes and the promotions of lipolysis in white adipose tissues [39]. It is speculated that the *ADRB3* rs4994 polymorphism affects binding to noradrenalin and coupling to G proteins in adipose cells [19]. *ADRB3* C allele carriers were shown to have a resting metabolic rate that was approximately 200 kcal lower [17] and lower lipolytic activity [23] in comparison to subjects with the T/T genotype. The findings of the current study suggest that the *ADRB3* C/C genotype is associated with not only an increased BMI and insulin resistance but also the risk of developing NAFLD indirectly (Figure 3).

Molecular abnormalities in ADRB3 are related to the development of obesity and type 2 diabetes [11, 40]. However, the association of the *ADRB3* rs4994 (i.e., Trp64Arg) polymorphism with overweight as well as insulin resistance is controversial and likely dependent on ethnicity. In addition, previous *in vitro* and *ex vivo* experiments suggested that the *ADRB3* Trp64Arg variant might affect the functional responsiveness, but several studies showed conflicting results [41]. Meanwhile, the *ADRB3* rs4994 (190T>C) polymorphism is associated with lower lipolytic activities in adipocytes [23], suggesting that this polymorphism may be associated with the accumulation of triglycerides in adipocytes, thereby leading to the accumulation of visceral fat and the development of insulin resistance [22, 23]. A recent clinical study of Spanish patients indicated that the polymorphism has a weak but significant influence on carriers leading to an increased fat mass and percentage [42]. More recently, in a larger patient cohort of Saudis patients, the polymorphism was linked to dyslipidemia and body weight gain [43]. Furthermore, recent meta-analyses showed that the *ADRB3* rs4994 polymorphism is significantly associated with the BMI and type 2 diabetes, especially in Asians, including the Japanese [18, 20, 21]. Therefore, the *ADRB3*rs4994 polymorphism is suspected to be associated with a tendency toward weight gain and insulin resistance [44], at least in East Asians. Based on the information, this study investigated the genetic effects in Japanese subjects and confirmed that individuals with the C/C genotype tended to be associated with a high BMI and HOMA-IR values compared to other genotypes among overweight/obese subjects (Table 6). The copresence of metabolic abnormalities associated with the rs4994 C allele is widely considered to be the pathogenesis of NAFLD [24, 25]. Indeed, insulin resistance in adipose tissue can dysregulate adipose tissue lipolysis and consequently increase the flux of fatty acids from adipocytes to the liver [26] and insulin resistance-induced hyperglycemia/hyperinsulinemia promotes hepatic *de novo* lipogenesis [27], both of which lead to the development of NAFLD [24, 25].

The current cross-sectional study suggests that individuals with the C/C genotype have a higher risk of NAFLD in comparison to those with other genotypes in overweight/obese (i.e., individuals with a BMI ≥ 23 kg/m^2^) but not normal-weight subjects (Tables 3 and 8). This study also revealed that the association between an increase in BMI and the presence of NAFLD might be more pronounced in the C/C genotype than in other genotypes (Figures 1 and 2). Furthermore, the longitudinal finding of this study suggested that overweight or obese individuals with the C/C genotype are predisposed to develop NAFLD compared with other individuals (Figure 4, Table 9). In addition, our structural equation model showed that a relationship between a high BMI and the risk of NAFLD existed in overweight/obese subjects (Figure 3) but not in normal-weight subjects. These results suggest that the C/C genotype might promote the development of NAFLD in overweight/obese individuals. Although this polymorphism might not be vital for the development of obese or NAFLD, the present findings are in line with the previous findings described above, so our results provide novel insight into the clinical significance of the *ADRB3* rs4994 polymorphism concerning NAFLD development.

Insulin resistance is a crucial pathophysiological factor in the development of NAFLD and a key mediator leading to further hepatic lipid accumulation and fibrogenic responses [25, 45]. The *ADRB3* rs4994 polymorphism has also been reported to be associated with insulin resistance [16]. The current study confirmed that among overweight/obese subjects, individuals with the C/C genotype tended to exhibit higher BMI, fasting serum insulin, and HOMA-IR values in comparison to other genotypes (Table 4). These results are in line with the findings of a previous study of patients with NASH, which showed that rs4994 C allele carriers had hyperinsulinemia as well as higher BMI and visceral fat levels in comparison to those with the T/T genotype [29]. Meanwhile, only *ADRB3* rs4994 C allele carriers with a BMI of ≤24.2 kg/m^2^ showed elevated ALT levels [28]. The results of our ROC curve analyses revealed that the cut-off BMI level associated with the presence of NAFLD in subjects with the C/C genotype was 23.0 kg/m^2^ (Figure 1). The present structural equation model revealed that the C/C genotype was associated with the latent variable in overweight/obese subjects only, which may reflect the reduced thermogenesis [17] and/or lipolysis abilities [23] in adipose tissues (Figure 3). This genotype was therefore indirectly associated with the risk of NAFLD through a high BMI and HOMA-IR (Figure 3). The findings from the current study thus suggest that the C/C genotype may accelerate weight gain as well as insulin resistance, which concertedly increase the accumulation of hepatic fat, resulting in the increased risk of NAFLD even in moderately obese individuals (i.e., those with a BMI of ≥23.0 kg/m^2^ among Japanese).

To date, several studies of the effects of lifestyle interventions among obese individuals have revealed that rs4994 C allele carriers show less weight reduction in comparison to other obese subjects [17, 46, 47], although several studies have shown conflicting results [48, 49]. Our results suggest that in combination with the strict weight control (e.g., aimed at achieving a BMI of <23 kg/m^2^ in Japanese individuals), *ADRB3* rs4994 polymorphism genotyping may provide a useful information to reduce the risk of developing NAFLD in individuals with the C/C genotype.

We investigated the association between ADRB3 genotype and NAFLD while paying careful attention to the BMI. The World Health Organization proposed a criterion for classifying body fat based on the BMI, whereby individuals with a BMI of ≥30 kg/m^2^ are classified as obese and those with a BMI of ≥25 kg/m^2^ are classified as overweight [30]. Recently, the American Diabetes Association changed the cut-off BMI for recommending the screening of Asian Americans for type 2 diabetes from 25 kg/m^2^ to 23 kg/m^2^ as well as from 30 kg/m^2^ to 27.5 kg/m^2^, based on evidence that shows that many Asian Americans develop the disease at lower BMI levels than the general population [31]. Thus, the difference in BMI cut-off levels among races should be carefully considered when the relationship between the BMI and the risk of NAFLD is investigated, and normal-weight, overweight, and obese statuses were defined as a BMI < 23 kg/m^2^, ≥23 kg/m^2^ to <27.5 kg/m^2^, and ≥27.5 kg/m^2^, respectively, in the current study.

In this study, the prevalence of obesity in subjects with the C/C genotype tended to be higher than that in T allele carriers, while the BMI values and the prevalence of overweight did not differ substantially between the genotypes in the overall study population (Table 1). The association between the rs4994 polymorphism and obesity is still controversial, and this polymorphism seems to have a weak effect on the actual BMI value [18]. Mice that lack *β*-adrenergic receptors (*β*-less mice) were reported to show a reduced metabolic rate, and *β*-less mice developed massive obesity on a high-fat diet but only slight obesity on a chow diet [50]. The polymorphism may therefore contribute to the capacity to gain weight in persons with an increased risk of developing obesity due to other, possibly additive, genetic, environmental, and behavioral factors [51]. Taken together, the C/C genotype may promote weight gain, especially in individuals who have already gained weight due to other environmental and behavioral factors (e.g., unhealthy lifestyle).

The present study is associated with several limitations. The association between the *ADRB3* genotype and the risk of NAFLD was retrospectively investigated in a relatively small number of subjects. Particularly, we could not divide the study subjects into overweight and obese subjects in all analyses because of the small sample size. To address this limitation, we validated the findings using a replicated data set consisting of 5,000 random samples from original data sets. However, even in the analysis containing 5,000 samples, the number of individuals with the C/C genotype was still small compared to those with other genotypes. Based on the results of our ROC curve analyses, the AUC values regarding the association between an increase in the BMI and the presence of NAFLD were significantly smaller in a replicated data set consisting of 5,000 random samples than in the original data sets (Figures 1 and 2). Furthermore, we only included Japanese health screening program participants, and the replicated data set consists of the data derived from the original data set; thus, it is possible that the results of the present study were affected by a selection bias, and it is unclear whether the present results can be generalized to other populations. Meanwhile, the subjects' alcohol consumption was evaluated through face-to-face interviews, which might have lacked reliability. Furthermore, NAFLD was diagnosed based on hepatic ultrasonography finding and was not confirmed by a liver biopsy. Finally, we were unable to consider the effects of lifestyle changes, such as physical activity, nutrition, or weight changes in our longitudinal analysis, which might have influenced the longitudinal association among the *ADRB3* polymorphism, weight status, and risk of NAFLD.

## 5. Conclusions

In conclusion, this cross-sectional and longitudinal study revealed that the *ADRB3* rs4994 polymorphism is associated with the development of NAFLD in overweight/obese subjects. The present findings provide novel insight into the clinical significance of the *ADRB3* rs4994 polymorphism concerning NAFLD development and may therefore accelerate the introduction of further preventive measures against NAFLD based on a patient's genetic background. The findings of this exploratory investigation warrant further large longitudinal or interventional studies, incorporating lifestyle changes, in larger and more diverse populations to verify the usefulness of *ADRB3* genotyping in the prevention of NAFLD in the general population.

## Figures and Tables

**Table 1:** The clinical characteristics of the overall study population.

	*ADRB3* rs4994 genotype			
	T/T (*n* = 366)	T/C (*n* = 202)	C/C (*n* = 23)	*p* value
Male/female	211/155	125/77	14/9	0.63^†^
Age (years)	67 (25–89)	66 (31–87)	69 (51–81)	0.21
BMI (kg/m^2^)	22.7 (15.5–34.7)	23.0 (15.9–37.2)	22.9 (17.2–35.8)	0.84
Overweight (%)	42.1	44.1	34.8	0.68^†^
Obesity (%)	4.9	6.4	13.0	0.17^†^
Alcohol intake (%)	44.0	46.5	52.2	0.68^†^
Ever smoking (%)	34.2	37.6	47.8	0.12^†^
Systolic BP (mmHg)	120 (75–182)	118.5 (93–177)	129 (94–163)	0.38
Diastolic BP (mmHg)	71 (44–102)	73 (50–102)	71 (59–93)	0.13
LDL_C (mg/dL)	120 (57–199)	122 (38–191)	128 (74–169)	0.55
HDL_C (mg/dL)	65 (34–119)	66 (30–128)	64 (42–114)	0.87
Triglyceride (mg/dL)	88 (26–520)	95 (31–431)	90 (45–210)	0.21
AST (IU/L)	22 (12–67)	22 (13–65)	23 (18–83)	0.34
ALT (IU/L)	20 (7–91)	20 (7–74)	21 (11–168)	0.39
GGT (IU/L)	22 (8–302)	23 (6–177)	27 (12–116)	0.25
HbA1c (%)^∗^	5.4 (4.8–9.8)	5.3 (3.8–8.7)	5.2 (4.9–7.4)	0.20
Fasting plasma glucose (mg/dL)	96 (68–244)	95 (73–206)	98 (78–131)	0.68
Fasting serum insulin (*μ*U/mL)^∗∗^	4.3 (0.7–33.3)	4.6 (1.1–22.3)	4.2 (1.9–38.6)	0.50
HOMA-IR^∗∗^	1.1 (0.1–8.3)	1.1 (0.2–6.4)	1.1 (0.4–10.9)	0.40
Hypertension (%)	38.5	38.6	47.8	0.67^†^
Diabetes (%)	13.7	12.9	13.0	0.98^†^
Dyslipidemia (%)	54.9	58.4	52.2	0.67^†^
NAFLD (%)	18.3	22.3	30.4	0.23^†^

Data are the median (range) or proportion for categorical variables. ADRB3: beta-3-adrenergic receptor; BMI: body mass index; BP: blood pressure; LDL_C: low-density lipoprotein cholesterol; HDL_C: high-density lipoprotein cholesterol; AST: aspartate transaminase; ALT: alanine transaminase; GGT: *γ*-glutamyltransferase; HbA1c: hemoglobin A1c; HOMA-IR: homeostasis model assessment for insulin resistance; NAFLD: nonalcoholic fatty liver disease. ^∗^Calculated among 384 subjects for whom information could be collected. ^∗∗^Calculated among 548 subjects for whom information could be collected. ^†^Analyzed by Fisher's exact test, otherwise Kruskal Wallis test.

**Table 2:** The association between the *ADRB3* genotype and the risk of NAFLD in the overall study population.

	Bivariable		Multivariable	
	OR [95% CI]	*p* value^∗^	OR [95% CI]	*p* value^∗^
*ADRB3* rs4994 genotype				
T/T	1	—	1	—
T/C	1.28 [0.84-1.96]	0.26	1.53 [0.91-2.57]	0.11
C/C	1.95 [0.77-4.93]	0.16	2.31 [0.67-8.01]	0.19
Age	0.98 [0.96-0.99]	<0.01	0.97 [0.95-0.99]	<0.01
Gender				
Male	1	—		
Female	0.57 [0.37-0.87]	<0.01		
BMI	1.55 [1.41-1.71]	<0.01	1.42 [1.27-1.58]	<0.01
HOMA-IR	2.22 [1.75-2.81]	<0.01	1.73 [1.35-2.23]	<0.01
Diabetes				
Absent	1	—		
Present	2.54 [1.52-4.25]	<0.01		
Dyslipidemia				
Absent	1	—	1	—
Present	4.21 [2.58-6.87]	<0.01	3.69 [2.06-6.61]	<0.01
Alcohol intake				
Nondrinker	1	—		
Drinker	0.97 [0.65-1.45]	0.88		
Smoking status				
Never smoker	1	—		
Ever smoker	1.58 [1.05-2.38]	0.03		

ADRB3: beta-3-adrenergic receptor; NAFLD: nonalcoholic fatty liver disease; BMI: body mass index; OR: odds ratio; CI: confidence interval. ^∗^Analyzed using a logistic regression analysis.

**Table 3:** The association between the *ADRB3* rs4994 genotype and the risk of NAFLD in normal-weight and overweight/obese subjects.

Weight status	*ADRB3* genotype	NAFLD^∗^		OR [95% CI]^∗∗^	*p* value^†^
		Absent	Present		
Normal-weight	T/T	184 (94.8)	10 (5.2)	1	
	T/C	92 (92.0)	8 (8.0)	1.92 [0.67–5.51]	0.18
	C/C	12 (100.0)	0 (0.0)	—	—

Overweight/obese	T/T	115 (66.9)	57 (33.1)	1	
	T/C	65 (63.7)	37 (36.3)	1.29 [0.73–2.29]	0.39
	C/C	4 (36.4)	7 (63.6)	4.40 [1.08–17.93]	0.04

Overweight	T/T	107 (69.5)	47 (30.5)	1	
	T/C	61 (68.5)	28 (31.5)	1.17 [0.63–2.19]	0.62
	C/C	3 (37.5)	5 (62.5)	6.18 [1.25–30.64]	0.03

ADRB3: beta-3-adrenergic receptor; NAFLD: nonalcoholic fatty liver disease; OR: odds ratio; CI: confidence interval. ^∗^Data are the number (%). ^∗∗^Adjusted by age, HOMA-IR, and dyslipidemia. ^†^Analyzed using a logistic regression analysis.

**Table 4:** The clinical characteristics of the overweight/obese subjects.

	*ADRB3* rs4994 genotype			
	T/T (*n* = 172)	T/C (*n* = 102)	C/C (*n* = 11)	*p* value
Male/female	114/58	74/28	8/3	0.56^†^
Age (years)	65 (29–88)	66 (31–83)	68 (51–80)	0.70
BMI (kg/m^2^)	25.0 (23.0–34.7)	24.7 (23.0–37.2)	25.0 (23.0–35.8)	0.63
Obesity (%)	4.1	2.0	27.3	<0.01^†^
Alcohol intake (%)	47.1	52.0	54.5	0.52^†^
Ever smoking (%)	40.7	45.1	54.5	0.56^†^
Systolic BP (mmHg)	121 (90–171)	120 (93–170)	121 (99–142)	0.81
Diastolic BP (mmHg)	74 (48–96)	75 (50–102)	71 (59–91)	0.25
LDL_C (mg/dL)	122 (61–199)	127 (76–191)	130 (108–169)	0.63
HDL_C (mg/dL)	57 (34–103)	59 (30–117)	62 (42–84)	0.90
Triglyceride (mg/dL)	104 (46–508)	110 (43–431)	122 (56–210)	0.36
AST (IU/L)	23 (14–67)	23 (13–65)	22 (18–83)	0.90
ALT (IU/L)	22 (7–91)	21 (10–74)	25 (15–168)	0.88
GGT (IU/L)	27 (10–180)	29 (6–163)	33 (20–116)	0.55
HbA1c (%)^∗^	5.4 (4.9–9.8)	5.4 (4.9–8.7)	5.2 (5.0–5.8)	0.45
Fasting plasma glucose (mg/dL)	98 (68–244)	96 (78–201)	98 (88–114)	0.82
Fasting serum insulin (*μ*U/mL)^∗∗^	5.6 (1.2–31.5)	5.2 (1.5–22.3)	6.7 (3.2–38.6)	0.43
HOMA-IR^∗∗^	1.4 (0.3–8.3)	1.2 (0.3–6.4)	1.6 (0.8–10.9)	0.45
Hypertension (%)	46.5	44.1	54.5	0.77^†^
Diabetes (%)	18.0	13.7	18.2	0.64^†^
Dyslipidemia (%)	65.1	70.6	63.6	0.65^†^
NAFLD (%)	33.1	36.3	63.6	0.12^†^

Data are the median (range) or proportion for categorical variables. ADRB3: beta-3-adrenergic receptor; BMI: body mass index; BP: blood pressure; LDL_C: low-density lipoprotein cholesterol; HDL_C: high-density lipoprotein cholesterol; AST: aspartate transaminase; ALT: alanine transaminase; GGT: *γ*-glutamyltransferase; HbA1c: hemoglobin A1c; HOMA-IR: homeostasis model assessment for insulin resistance; NAFLD: nonalcoholic fatty liver disease. ^∗^Calculated among 170 subjects for whom information could be collected. ^∗∗^Calculated among 267 subjects for whom information could be collected. ^†^Analyzed by Fisher's exact test, otherwise Kruskal Wallis test.

**Table 5:** The clinical characteristics of the normal-weight subjects.

	*ADRB3* rs4994 genotype			
	T/T (*n* = 194)	T/C (*n* = 100)	C/C (*n* = 12)	*p* value
Male/female	97/97	51/49	6/6	0.98^†^
Age (years)	69 (25–89)	67 (31–87)	70 (54–81)	0.15
BMI (kg/m^2^)	20.9 (15.5–22.9)	20.8 (15.9–22.9)	21.7 (17.2–22.9)	0.62
Alcohol intake (%)	41.2	41.0	50.0	0.83^†^
Ever smoking (%)	28.4	30.0	41.7	0.55^†^
Systolic BP (mmHg)	118 (75–182)	118 (98–177)	130 (94–163)	0.19
Diastolic BP (mmHg)	69 (44–102)	70 (50–96)	71 (64–93)	0.15
LDL_C (mg/dL)	118 (57–188)	118 (38–178)	128 (74–145)	0.85
HDL_C (mg/dL)	72 (39–119)	74 (35–128)	68 (45–114)	0.59
Triglyceride (mg/dL)	78 (26–520)	85 (31–349)	87 (45–172)	0.68
AST (IU/L)	22 (12–39)	22 (14–34)	24 (18–46)	0.26
ALT (IU/L)	17 (7–51)	18 (7–71)	19 (11–36)	0.34
GGT (IU/L)	18 (8–302)	21 (8–177)	20 (12-57)	0.27
HbA1c (%)^∗^	5.4 (4.8–7.6)	5.3 (3.8–8.4)	5.3 (4.9–7.4)	0.26
Fasting plasma glucose (mg/dL)	94 (74–162)	95 (73–206)	94 (78–131)	0.51
Fasting serum insulin (*μ*U/mL)^∗∗^	3.4 (0.7–33.3)	4.0 (1.1–19.0)	3.1 (1.9–9.5)	0.17
HOMA-IR^∗∗^	0.8 (0.1–7.8)	1.0 (0.2–5.2)	0.7 (0.4–3.1)	0.06
Hypertension (%)	31.4	33.0	41.7	0.72^†^
Diabetes (%)	9.8	12.0	8.3	0.83^†^
Dyslipidemia (%)	45.9	46.0	41.7	1.00^†^
NAFLD (%)	5.2	8.0	0.0	0.52^†^

Data are the median (range) or proportion for categorical variables. ADRB3: beta-3-adrenergic receptor; BMI: body mass index; BP: blood pressure; LDL_C: low-density lipoprotein cholesterol; HDL_C: high-density lipoprotein cholesterol; AST: aspartate transaminase; ALT: alanine transaminase; GGT: *γ*-glutamyltransferase; HbA1c: hemoglobin A1c; HOMA-IR: homeostasis model assessment for insulin resistance; NAFLD: nonalcoholic fatty liver disease. ^∗^Calculated among 214 subjects for whom information could be collected. ^∗∗^Calculated among 281 subjects for whom information could be collected. ^†^Analyzed by Fisher's exact test, otherwise Kruskal Wallis test.

**Table 6:** The association between the *ADRB3* rs4994 genotype and the BMI or HOMA-IR values in normal-weight and overweight/obese subjects.

Dependent variables	Weight status	*ADRB3* genotype^∗^	*B* ^∗∗^	SE	*p* value^†^
BMI	Normal-weight	T/T (194)	0		
		T/C (100)	0.06	0.20	0.75
		C/C (12)	0.39	0.48	0.43
	Overweight/obese	T/T (172)	0		
		T/C (102)	-0.14	0.28	0.62
		C/C (11)	1.43	0.68	0.04

HOMA-IR	Normal-weight	T/T (181)	0		
		T/C (89)	0.13	0.10	0.18
		C/C (11)	0.07	0.23	0.77
	Overweight/obese	T/T (159)	0		
		T/C (97)	-0.09	0.16	0.62
		C/C (11)	0.78	0.38	0.04

ADRB3: beta-3-adrenergic receptor; SE: standard error. ^∗^Data are the number (%). ^∗∗^Adjusted by age, diabetes, and dyslipidemia. ^†^Analyzed using a multiple linear regression analysis.

**Table 7:** The clinical characteristics of the samples using by 5,000 randomly sampled data sets.

	*ADRB3* rs4994 genotype			
	T/T (*n* = 3084)	T/C (*n* = 1706)	C/C (*n* = 210)	*p* value
Age (years)	66 (17–97)	65 (20–93)	66 (34–91)	0.41
BMI (kg/m^2^)	22.8 (14.1–35.2)	22.8 (14.5–33.7)	22.9 (14.6–32.9)	0.96
Diabetes (%)	13.4	13.4	10.5	0.49^†^
Dyslipidemia (%)	55.8	60.1	48.1	< 0.01^†^
NAFLD (%)	18.1	22.5	28.6	< 0.01^†^

Data are the median (range) or proportion for categorical variables. ADRB3: beta-3-adrenergic receptor; BMI: body mass index; NAFLD: nonalcoholic fatty liver disease. ^†^Analyzed by Fisher's exact test, otherwise Kruskal Wallis test.

**Table 8:** The association between the *ADRB3* rs4994 genotype and the risk of NAFLD in normal-weight and overweight/obese subjects, as determined using 5,000 randomly sampled data sets.

Weight status	*ADRB3* genotype	NAFLD^∗^		OR [95% CI]^∗∗^	*p* value^†^
		Absent	Present		
Normal-weight	T/T	1357 (82.3)	292 (17.7)	1	
	T/C	687 (76.5)	211 (23.5)	1.38 [1.12–1.70]	<0.01
	C/C	88 (80.7)	21 (19.3)	1.38 [0.82–2.32]	0.22

Overweight/obese	T/T	1169 (81.5)	266 (18.5)	1	
	T/C	635 (78.6)	173 (21.4)	1.16 [0.93–1.46]	0.183
	C/C	62 (61.4)	39 (38.6)	3.26 [2.08–5.12]	<0.001

Overweight	T/T	990 (81.2)	229 (18.8)	1	
	T/C	538 (78.7)	146 (21.3)	1.14 [0.89–1.45]	0.30
	C/C	52 (59.8)	35 (40.2)	3.76 [2.31–6.13]	<0.01

ADRB3: beta-3-adrenergic receptor; NAFLD: nonalcoholic fatty liver disease; BMI: body mass index; OR: odds ratio; CI: confidence interval; NAFLD: nonalcoholic fatty liver disease. ^∗^Data are the number (%). ^∗∗^Adjusted by age, BMI, diabetes, and dyslipidemia. ^†^Analyzed using a logistic regression analysis.

**Table 9:** Association between the risk of NAFLD during the observation period (year) and the combination of weigh status at baseline and the *ADRB3* rs4994 genotype in the Cox proportional hazard model.

Weight status	*ADRB3* genotype	HR [95% CI]^∗^	*p* value^∗∗^
Normal-weight	T allele	1 3.66 [0.45–30.15]	0.23
	C/C		

Overweight/obese	T allele	3.53 [1.42–8.79]	<0.01
	C/C	10.03 [2.04–49.37]	<0.01

ADRB3: beta-3-adrenergic receptor; NAFLD: nonalcoholic fatty liver disease; HR: hazard ratio; CI confidence interval. ^∗^Adjusted by age, HOMA-IR, and dyslipidemia. ^∗∗^Analyzed using a Cox proportional hazard model.

## Data Availability

The data used to support the findings of this study are available from the corresponding author upon request.
